# Placas Vulneráveis Detectadas por TC Cardíaca e Risco de Infarto Agudo do Miocárdio Futuro: Valor Preditivo dos Escores CAC e do Índice de Carga Inflamatória

**DOI:** 10.36660/abc.20250052

**Published:** 2025-12-08

**Authors:** Sefa Erdi Ömür, Emin Koyun, Gökhan Cabri

**Affiliations:** 1 Department of Cardiology Tokat Gaziosmanpaşa University Tokat Turquia Department of Cardiology, Tokat Gaziosmanpaşa University, Tokat – Turquia; 2 Cardiology Clinic Sivas State Hospital Sivas Turquia Cardiology Clinic, Sivas State Hospital, Sivas – Turquia

**Keywords:** Vasos Coronários, Infarto do Miocárdio, Angiografia por Tomografia Computadorizada

## Abstract

**Fundamento:**

A angiotomografia computadorizada (ATC) é amplamente utilizada como modalidade básica não invasiva para avaliar a doença arterial coronariana. O índice de carga inflamatória (IBI *Inflammatory burden index* ) é um indicador de inflamação sistêmica que reflete o estado inflamatório. Ainda é difícil determinar o alto risco de infarto agudo do miocárdio (IAM) futuro em pacientes com placa detectada pela angiotomografia.

**Objetivo:**

Este estudo teve como objetivo avaliar a eficácia dos escores de Cálcio da Artéria Coronária (CAC) e IBI na predição do risco de IAM entre pacientes submetidos à ATC para dor torácica. Buscamos avaliar o valor preditivo individual e combinado desses índices, determinar seus valores de corte ideais e comparar o risco de IAM entre grupos de pacientes com diferentes escores CAC e IBI. Também objetivamos avaliar o papel potencial desses marcadores na identificação de “pacientes vulneráveis” com alto risco de eventos cardiovasculares futuros.

**Métodos:**

Este estudo retrospectivo incluiu 1.235 pacientes submetidos à ATC devido à dor torácica e acompanhados por 3 anos (2,8-3,4). Os pacientes foram categorizados em três modelos com base em seus escores CAC e IBI. O desfecho primário foi a ocorrência de IAM durante o período de acompanhamento. A significância estatística foi estabelecida em p < 0,05 para todas as análises.

**Resultados:**

Pacientes que apresentaram IAM apresentaram escores de IBI e CAC significativamente mais altos em comparação com aqueles sem ISM. A análise de regressão logística identificou o escore de CAC e o IBI como preditores independentes de ISM. A análise ROC determinou os valores de corte ideais para IBI (128) e CAC (102) na predição de IDM. A análise de Kaplan-Meier revelou um gradiente significativo no risco de IAM entre os três modelos, com o maior risco observado em pacientes com altos escores de IBI e CAC.

**Conclusão:**

A combinação dos escores IBI e CAC proporciona melhor estratificação de risco para a previsão de IAM futuro (placa vulnerável) em pacientes com placa detectada por angiotomografia. Essa abordagem pode facilitar estratégias de tratamento mais específicas para pacientes de alto risco.

## Graphical abstract

**Figura Central:**
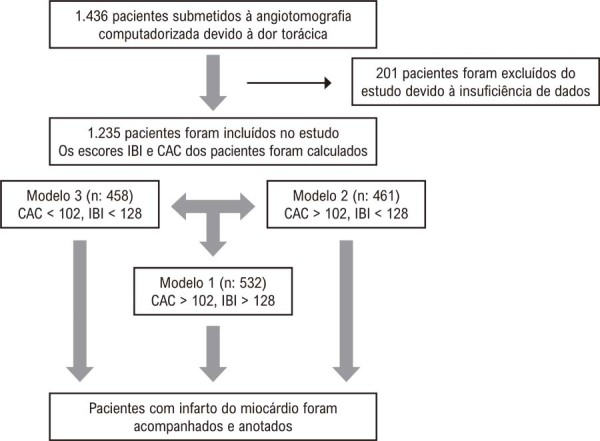
Placas Vulneráveis Detectadas por TC Cardíaca e Risco de Infarto Agudo do Miocárdio Futuro: Valor Preditivo dos Escores CAC e do Índice de Carga Inflamatória

Fluxograma do estudo.

## Introdução

O infarto agudo do miocárdio (IAM) continua sendo uma das principais causas de morbidade e mortalidade em todo o mundo. Apesar dos avanços nas técnicas diagnósticas e intervenções terapêuticas, a identificação e a previsão precoces do IAM, particularmente em pacientes com dor torácica, continuam a representar desafios significativos para os médicos. A angiotomografia computadorizada (ATC) emergiu como uma ferramenta crucial na avaliação não invasiva da doença arterial coronariana (DAC) e na caracterização de placas, fornecendo informações valiosas sobre a presença e a extensão da aterosclerose coronariana, bem como a identificação de placas vulneráveis e propensas à ruptura.^1^

A inflamação desempenha um papel fundamental na patogênese da aterosclerose e na desestabilização da placa, levando ao IAM.^2^ Nos últimos anos, assistimos a uma mudança de paradigma na medicina cardiovascular, passando de um foco exclusivo em placas vulneráveis para um conceito mais abrangente de paciente vulnerável. O paciente vulnerável abrange não apenas as características locais da placa, mas também fatores como inflamação sistêmica e carga aterosclerótica generalizada.^3^ Nosso estudo explora como a combinação de marcadores sistêmicos, como o Escore de Cálcio da Artéria Coronária (CAC) e o Índice de Carga Inflamatória (IBI), pode ajudar a identificar não apenas placas vulneráveis, mas também pacientes vulneráveis e suscetíveis a futuros eventos cardiovasculares. Biomarcadores de inflamação, como a proteína C-reativa (PCR) e a razão neutrófilo-linfócito (NLR), foram reconhecidos como valiosos preditores de eventos cardiovasculares.^4^ Estudos recentes examinaram a relação entre a carga inflamatória e o potencial para doenças cardiovasculares. Pesquisadores combinaram PCR e NLR para criar a medida IBI, que agora é amplamente utilizada em estudos de prognóstico de câncer e doenças cerebrovasculares.^5 , 6^ Um estudo recente encontrou uma associação entre os níveis de IBI e a prevalência de doenças cardiovasculares.^7^ Comparado à PCR e à NLR, o IBI, um marcador abrangente de inflamação, pode fornecer uma avaliação mais consistente da inflamação, refletir com mais precisão o estado inflamatório do corpo, avaliar melhor a eficácia do tratamento e prever o prognóstico dos pacientes.^8^ O Sistema de Notificação e Dados de Doença Arterial Coronária (CAD-RADS *Coronary Artery Disease-Reporting and Data System* ) tem demonstrado que prevê com precisão os principais eventos cardiovasculares adversos, definidos como angina instável, IAM ou morte, em pacientes com dor torácica estável, com desempenho superior em comparação aos fatores de risco tradicionais, outros escores de estratificação de risco, o CAC e o sistema anterior de escore de estenose coronária por tomografia computadorizada cardiovascular.^9 , 10^

Além disso, o escore CAC, uma medida bem estabelecida da carga da placa aterosclerótica, tem sido amplamente utilizado para estratificar o risco em pacientes com DAC.^11^ No entanto, a contribuição relativa dos escores CAC e IBI na predição da erosão da placa, que é um precursor do IM, ainda não está totalmente esclarecida. Compreender a relação entre esses índices e sua capacidade de prever a instabilidade da placa pode impactar significativamente na tomada de decisões clínicas e o manejo do paciente.^12^

Este estudo tem como objetivo avaliar a eficácia dos escores CAC e IBI na predição de erosão de placa e subsequente IAM em pacientes que apresentam dor torácica e são submetidos à angiotomografia. Ao examinar retrospectivamente esses fatores em uma grande coorte, este estudo busca fornecer insights sobre sua utilidade para aprimorar a estratificação de risco e orientar intervenções terapêuticas no ambiente cardiológico ambulatorial.

## Métodos

### População do estudo e coleta de dados

Esta investigação de coorte prospectiva foi conduzida na clínica de cardiologia de um hospital universitário local. A população do estudo consistiu em pacientes que apresentaram angina de peito estável ou dor torácica atípica e foram submetidos a ATC diagnóstica entre junho de 2019 e junho de 2021. O estudo incluiu pacientes que não apresentavam angina instável no início do estudo. Durante o período de acompanhamento, alguns pacientes desenvolveram IAM enquanto outros permaneceram livres de eventos. Todos os pacientes foram monitorados para eventos de IAM durante todo o período do estudo. Dos 1235 pacientes incluídos na análise final, 283 pacientes apresentaram IAM durante o acompanhamento, enquanto 952 pacientes permaneceram livres de eventos de IAM. Os pacientes incluídos no estudo apresentaram valores iniciais de troponina dentro da faixa normal e nenhuma alteração típica em seus eletrocardiogramas sugestivos de infarto por IAM. Os tratamentos médicos (ácido acetilsalicílico, terapia anti-hiperlipidêmica, etc.) dos pacientes foram otimizados após a ATC. Todos os pacientes incluídos no estudo foram acompanhados prospectivamente para eventos de IAM até junho de 2024, resultando em um acompanhamento médio de 3 (2,8-3,4) anos.

As imagens ATC foram analisadas para identificar placas, com foco específico em lesões que demonstravam estenose inferior a 50%. Entre os pacientes que apresentaram IAM durante o período de acompanhamento, suas ATC basais foram avaliadas retrospectivamente para caracterizar as placas culpadas. As características vulneráveis das placas foram avaliadas sistematicamente usando critérios estabelecidos de imagem por tomografia computadorizada. A avaliação incluiu a medição quantitativa da atenuação da placa, com placas de baixa atenuação definidas como aquelas com < 30 unidades Hounsfield. A remodelação positiva foi identificada por um índice de remodelação superior a 1,1, calculado como a razão entre o diâmetro do vaso no local da placa e o diâmetro de referência do segmento proximal normal. Outras características de alto risco da placa que foram avaliadas incluíram padrões de calcificação irregulares e a presença do sinal do anel de guardanapo (NRS). Esses marcadores de imagem por ATC validados foram analisados em conjunto com os escores CAC e IBI para fornecer uma avaliação abrangente da vulnerabilidade da placa. Todas as imagens de TC foram avaliadas independentemente por dois radiologistas cardiovasculares experientes, cegos aos desfechos clínicos, com quaisquer discrepâncias resolvidas por consenso.

Inicialmente, 1.436 pacientes preencheram os critérios de inclusão; no entanto, 201 foram excluídos devido a dados incompletos ou recusa de participação ( Figura Central ), resultando em uma coorte final de estudo de 1.235 pacientes. As características demográficas, clínicas, médicas e angiográficas dos pacientes e os resultados de acompanhamento de 3 anos foram obtidos usando dados de pacientes hospitalares e banco de dados hospitalares. O estudo está em conformidade com os princípios estabelecidos na Declaração de Helsinque e foi aprovado pelo comitê de ética do hospital universitário local. A totalidade deste manuscrito foi escrita sem o uso de quaisquer tecnologias assistidas por inteligência artificial (IA), incluindo grandes modelos de linguagem, *chatbots* ou criadores de imagens. Todo o conteúdo foi produzido exclusivamente pelos autores usando métodos tradicionais de escrita e pesquisa.

Os desfechos dos pacientes foram monitorados sistematicamente por meio de um protocolo abrangente de acompanhamento multimodal. Todos os pacientes inscritos foram agendados para consultas clínicas regulares em nossa instituição, onde avaliações cardiovasculares detalhadas foram realizadas e documentadas. Para os pacientes que faltaram às consultas agendadas, entrevistas telefônicas estruturadas foram conduzidas com os pacientes ou seus parentes próximos designados, utilizando informações de contato do banco de dados do nosso hospital. Nos casos em que os pacientes relataram eventos de IAM em outras unidades de saúde, nossa equipe de pesquisa estabeleceu comunicação direta com os cardiologistas responsáveis pelo tratamento nessas instituições. Além disso, todos os eventos dos pacientes foram verificados por meio do sistema nacional de registros eletrônicos de saúde (e-Nabiz), que forneceu acesso a imagens angiográficas e dados clínicos abrangentes de qualquer unidade de saúde da rede nacional. Essa abordagem multifacetada para o monitoramento do paciente, combinando acompanhamento clínico direto, telecomunicação sistemática, consulta médica interinstitucional e revisão centralizada de registros eletrônicos de saúde, permitiu a documentação e a verificação completas dos eventos de IAM, independentemente do local de ocorrência.

Os critérios da Diretriz da Sociedade Europeia de Cardiologia foram usados para diagnosticar IAM em 13 pacientes. Pacientes que apresentaram IAM no ponto de placa indicado de acordo com o resultado da angiografia por TC (n: 283) e aqueles que não apresentaram (n: 952) foram incluídos no estudo, e os pacientes foram avaliados em termos de escores IBI e CAC. Três sistemas de modelagem foram criados para avaliar o sucesso dos escores IBI e CAC na predição da erosão da placa em pacientes. De acordo com esse sistema de modelagem, pacientes com altos escores CAC e IBI foram incluídos no grupo de pacientes no modelo 1. Pacientes com altos escores CAC e baixos valores IBI foram incluídos no grupo do modelo 2. Pacientes com baixos escores IBI e CAC foram incluídos no grupo do modelo 3. Os valores de CAC e IBI foram adicionados aos modelos como variáveis contínuas, de acordo com o valor de corte obtido na análise ROC. Os pacientes foram acompanhados por uma média de 3 (2,8-3,4) anos, de acordo com os três modelos, e as taxas de IM foram registradas. O escore CAD-RADS, que foi desenvolvido para padronizar os relatórios de imagens de TC coronárias, melhorar a comunicação e orientar o tratamento, foi avaliado e registrado para os pacientes. O IBI é definido como o produto da PCR multiplicada pela NLR. Pacientes com qualquer doença inflamatória sistêmica e reumatológica, doença de depósito, anemia, malignidade, idade inferior a 18 anos, acidente vascular cerebral agudo ou crônico, qualquer doença hematológica, incluindo insuficiência renal e/ou hepática avançada, pacientes com histórico de infecção aguda ou crônica, pacientes com histórico de transfusão de sangue nos últimos 3 meses, pacientes com doença valvar grave e pacientes submetidos a cirurgia valvar foram excluídos do estudo. Ao mesmo tempo, pacientes com estenose superior a 50% na angiotomografia não foram incluídos no estudo, pois foram encaminhados diretamente para angiografia.

### Exame laboratorial e demográfico

Todas as amostras de sangue foram obtidas de sangue venoso periférico imediatamente antes da angiotomografia. NT-proBNP, PCR, painel lipídico, glicemia de jejum, creatinina, troponina-I e outros parâmetros de rotina foram obtidos das amostras de sangue. O hemograma completo (HC) foi avaliado com um contador automático de células sanguíneas (Coulter LH 780 Hematology Analyzer, Beckman Coulter Corp, Hialeah, Flórida, EUA). Pacientes com nível de glicemia de jejum > 125 mg/dL, nível de HgA1c > 6,5% ou usando medicamentos antidiabéticos (orais/insulina) foram considerados pacientes com DM. Pacientes com níveis de colesterol de lipoproteína de baixa densidade (LDL-C) acima de 100 mg/dL ou usando medicamentos anti-lipidêmicos foram considerados pacientes hiperlipidêmicos (LH). O uso de medicamentos anti-hipertensivos ou pressões arteriais sistólica e diastólica acima de 140/90 mmHg foi considerado como hipertensão (HT). DAC foi definida como estenose significativa das artérias coronárias, estreitamento do diâmetro médio do lúmen superior a 50% ou evidência de infarto miocárdico prévio. Em nossa avaliação de pacientes com IAM, consideramos não apenas as características individuais da placa, mas também fatores sistêmicos que podem contribuir para o conceito geral de “paciente vulnerável”, indivíduos com risco elevado de eventos cardiovasculares devido a uma combinação de vulnerabilidade da placa, estado inflamatório e outros fatores sistêmicos. Pacientes que fumaram nos últimos seis meses foram considerados fumantes.

### Angiografia coronária, avaliação ecocardiográfica

Antes da angiografia coronária, todos os pacientes foram submetidos a ecocardiografia transtorácica (ETT) com aparelho de ecocardiografia Vivid E7 (GE Vingmed Ultrasound) e sonda de ultrassom MS5 (1,5-4,5 MHz) em decúbito esquerdo. A fração de ejeção do ventrículo esquerdo (FEVE) foi medida por dois cardiologistas experientes, utilizando o método de Simpson. Todos os procedimentos de CAG e ICP foram realizados com o dispositivo de angiografia Xper Allura FD-10 Modelo C Arm Detector System (Philips Medical Systems International BV, Best, Holanda). Todos os pacientes foram submetidos a intervenção femoral ou radial com a técnica padrão de Judkins e um cateter de 6 Fr. Dois cardiologistas intervencionistas experientes realizaram procedimentos de ICP.

### Análise estatística

As análises estatísticas foram realizadas usando o software SPSS 26.0 (SPSS Inc., Chicago, IL, EUA). A normalidade da distribuição dos dados foi avaliada usando o teste de Kolmogorov-Smirnov. Variáveis categóricas foram expressas como números e porcentagens (n, %), enquanto variáveis contínuas foram apresentadas como média ± desvio padrão (média ± DP) ou mediana com intervalo interquartil (mediana, IIQ) de acordo com seu padrão de distribuição. Para dados paramétricos que atendem às premissas necessárias (medidas de escala de intervalo/razão com distribuição normal), um teste-t de amostras independentes foi usado para comparar dois grupos independentes, enquanto ANOVA unidirecional foi empregada para comparar mais de dois grupos independentes. Ao usar ANOVA para comparações de múltiplos grupos, a análise post-hoc foi conduzida usando o teste de Tukey para variâncias homogêneas e o teste de Games-Howell para variâncias não homogêneas para determinar quais grupos diferiram significativamente dos outros. Quando as premissas paramétricas não foram atendidas, o teste U de Mann-Whitney foi usado para comparar dois grupos independentes. O teste chi-quadrado foi utilizado para analisar variáveis categóricas. A análise da curva (ROC) foi usada para calcular os melhores valores de corte de CAC e IBI. O melhor valor de corte obtido da curva ROC de CAC e IBI foi tomado como o valor de corte na categorização dos valores de escore de IBI e CAC como altos e baixos. A análise de regressão logística univariada foi realizada para determinar os preditores de IAM em todos os pacientes. As variáveis que foram significativas na análise de regressão logística univariada (p < 0,05) foram incluídas na análise de regressão logística multivariada. Os resultados da análise de regressão logística são apresentados como odds ratios (OR) e intervalos de confiança de 95% (IC). A curva de sobrevida de Kaplan-Meier foi usada para examinar a diferença nas taxas de incidência de IM entre os grupos, e a significância estatística foi determinada usando o teste Log-rank. Um P bilateral < 0,05 foi considerado estatisticamente significativo.

## Resultados

Um total de 1.235 pacientes submetidos à ATC foi incluído em nosso estudo. As características demográficas, clínicas e laboratoriais básicas, os resultados angiográficos e as medicações dos pacientes incluídos no estudo são apresentados na Tabela 1 . Doença arterial coronária, diabetes e hiperlipidemia foram encontradas em níveis mais elevados no grupo com IAM. Contagem de neutrófilos, plaquetas, colesterol LDL, triglicerídeos, PCR e valores de NT-proBNP foram significativamente maiores no grupo IM do que no grupo sem IM. Os níveis de troponina foram maiores no grupo IAM como esperado. A contagem de linfócitos foi maior no grupo sem IAM. Quando o valor do IBI foi examinado, foi encontrado significativamente maior no grupo com IAM do que no grupo sem IAM. CAC, uso de estatina e e/e’ foram maiores no grupo IAM em comparação com o grupo sem IAM.

**Tabela 1:** – Comparação dos dados demográficos, laboratoriais e medicamentos dos grupos

Variável	Infarto do miocárdio (n: 397)	Sem infarto do miocárdio (n: 952)	p
Idade (anos)	65,41±3,24	64,45±4,03	0,180
Sexo feminino n (%)	174 (43,82)	423 (44,43)	0,639
IMC kg/m^2^	27,43±3,22	26,91±3,14	0,221
Diabetes mellitus n (%)	138 (34,76)	298 (31,30)	**0,003**
Hipertensão n (%)	131 (32,99)	321 (33,71)	0,861
Hiperlipidemia n (%)	135 (34,00)	301 (31,61)	**0,017**
Tabagismo n (%)	140 (35,26)	326 (34,24)	0,283
Fibrilação atrial n (%)	47 (11,83)	111 (11,65)	0,569
DAC n (%)	127 (31,98)	298 (31,30)	**0,040**
DAP n (%)	23 (5,79)	52 (5,46)	0,079
CAD-RADS	2,2±0,53	1,6±0,21	**<0,001**
**Variáveis hematológicas**			
Glicose em jejum (mg/dl)	118,62±12,79	116,63±11,78	0,829
Creatinina (mg/dl)	1,01±0,41	0,99±0,55	0,129
Sódio (mmol/L)	138,55±3,76	139,66±2,86	0,873
Potássio (mmol/L)	4,50±0,32	4,51±0,34	0,499
ALT (U/L)	25,39±2,81	24,59±2,87	0,553
AST (U/L)	24,37±3,16	24,51±4,82	0,359
TSH (µUI/ml)	1,44±0,32	1,41±0,29	0,283
Hemoglobina (g/dl)	10,61±3,41	10,53±3,59	0,573
Contagem de leucócitos (x10^3^/µl)	10,11±3,11	9,28±4,07	0,449
Contagem de neutrófilos (×10^3^ /μL)	8,14±2,44	3,89±2,31	**<0,001**
Contagem de linfócitos (×10^3^ /μL)	1,11±0,74	1,29±1,94	**<0,001**
Monócitos (x10^3^ /µl)	1,85±1,51	1,84±1,31	0,113
Plaquetas (x10^3^/µl)	349,21±61,23	328,91±66,73	**<0,001**
Colesterol LDL (mg/dl)	136,44 ± 30,8	125,6 ± 41,12	**<0,001**
Colesterol HDL (mg/dl)	37,69±12,41	42,26±10,1	**0,048**
Triglicerídeos, (mg/dl)	231,81±32,21	218,51 ± 27,6	**0,039**
NT-proBNP (pg/ml)	52,41±12,32	44,2±10,7	**0,028**
Troponina (ng/mL)	99,53±29,36	57,24±25,68	**<0,001**
PCR (mg/L)	45,24±9,56	21,42±8,41	**<0,001**
IBI	326,88±32,57	64,49±15,21	**<0,001**
**Achados ecocardiográficos**			
FEVE (%)	52,41±2,6	52,51±2,9	0,947
Tamanho AE (mm)	35,51±4,8	34,42±5,01	0,557
IMVE (g/m)	112,32±2,3	111,2±3,6	0,469
**Parâmetros angiográficos**			
Lesão da DAE n(%)	166 (41,81)	398 (41,80)	0,783
Lesão CX n(%)	138 (34,76)	342 (35,92)	0,663
Lesão ACD n(%)	120 (30,22)	296 (31,09)	0,239
Doença de três vasos n(%)	20 (5,03)	51 (5,35)	0,570
Escore CAC	342±25,31	88±12,3	**<0,001**
**Medicamentos**			
Ácido acetilsalicílico	178 (44,83)	425 (44,62)	0,601
IECA, BRA	123 (30,98)	288 (30,25)	0,122
Betabloqueador	91 (22,92)	220 (23.10)	0,806
Estatina	111 (27,95)	218 (22,89)	**0,011**
Clopidogrel	16 (4,03)	43 (4,51)	0,347
Bloqueadores dos canais de cálcio	39 (9,82)	99 (10,39)	0,779

Valores em negrito indicam valores de p estatisticamente significativos (p<0,05). IECA: Inibidor da enzima conversora de angiotensina; ALT: alanina aminotransferase; BRA: bloqueador do receptor de angiotensina; AST: aspartato aminotransferase; IMC: índice de massa corporal; CAC: cálcio da artéria coronária; DAC: doença arterial coronária; CAD-RADS: sistema de notificação e dados de doença arterial coronária; PCR: Proteína C reativa; CX, artéria circunflexa; HDL: lipoproteína de alta densidade; IBI: índice de carga inflamatória; AE: átrio esquerdo; DAE: artéria descendente anterior esquerda; LDL: lipoproteína de baixa densidade; FEVE: fração de ejeção do ventrículo esquerdo; IMVE: índice de massa ventricular esquerda; NT-proBNP: pró-peptídeo natriurético cerebral N-terminal; DAP: doença arterial periférica; ACD: artéria coronária direita; TSH: hormônio estimulante da tireoide.

Quinhentos trinta e dois pacientes (36,66%) foram incluídos no grupo de pacientes no modelo 1, 461 pacientes (31,77%) foram incluídos no grupo de pacientes no modelo 2 e 458 pacientes (31,56%) foram incluídos no grupo de pacientes no modelo 3. A classificação dos pacientes de acordo com os escores IBI e CAC é mostrada na Tabela 2 . Doença arterial coronariana, diabetes e hiperlipidemia foram encontradas como sendo maiores no grupo do Modelo 1 em comparação aos outros dois grupos. Contagem de neutrófilos, plaquetas, PCR, colesterol LDL, triglicerídeos, NT-proBNP e troponina foram maiores no grupo Modelo 1 em comparação aos outros dois grupos. Na avaliação angiográfica, as taxas de lesão da DA, LCX e ACD foram semelhantes nos grupos Modelo 1 e Modelo 2, enquanto foram significativamente maiores nesses dois grupos em comparação ao grupo Modelo 3. Na avaliação ecocardiográfica, a relação e/e’ foi maior no grupo Modelo 1 em comparação aos outros dois grupos.

**Tabela 2:** – Comparação de dados demográficos, laboratoriais e medicamentos dos modelos

Variável	MODELO 1 (n: 532)	MODELO 2 (n: 461)	MODELO 3 (n: 458)	p
Idade (anos)	65,21±1,14	65,25±3,03	64,79±8,68	0,263
Sexo feminino n (%)	248 (46,61)	210 (45,55)	209 (45,63)	0,429
IMC kg/m^2^	27,23±4,22	28,01±3,84	28,03±4,74	0,367
Diabetes mellitus n (%)	172 (32,33)^a^	140 (30.36)^b^	136 (29,69)^c^	0,023
Hipertensão n (%)	161 (30,26)	132 (28,63)	135 (29,47)	0,351
Hiperlipidemia n (%)	167 (31,39)^a^	129(27.33)^b^	114(24,89)^c^	0,012
Tabagismo n (%)	128(24,06)	109(23,64)	107(23,36)	0,189
Fibrilação atrial n (%)	65 (12.21)	58 (12,58)	56 (12,22)	0,243
DAC n (%)	153 (28,95)^a^	132 (28,63)^b^	100 (21,83)^c^	**0,032**
DAP n (%)	35 (6,57)	28 (6,07)	26 (5,67)	0,089
Taxas de IM n (%)	202 (37,9)^a^	157 (34,05)^b^	38 (8,29)^c^	**<0,001**
CAD-RADS	2,3±0,41^a^	2,2±0,39^a^	1,3±0,12^b^	**<0,001**
**Variáveis hematológicas**				
Glicose em jejum (mg/dl)	119,46±11,83	117,77±12,68	118,43±14,6	0,751
Creatinina (mg/dl)	0,98±0,45	0,99±0,25	1,08±0,41	0,127
Sódio (mmol/L)	139,56±2,86	137,62±3,8	138,18±4,58	0,891
Potássio (mmol/L)	4,51±0,34	4,50±0,44	4,43±0,52	0,493
ALT (U/L)	22,28±2,95	21,23±3,96	19,14±5,1	0,351
AST (U/L)	23,44±3,16	22,76±6,92	22,31±5,01	0,379
TSH(µUI/ml)	1,59±0,28	1,55±0,34	1,52±0,44	0,283
Hemoglobina (g/dl)	10,61±2,31	10,69±2,19	11,18±2,74	0,124
Contagem de leucócitos (x10^3^/µl)	9,81±5,00	9,88±5,47	9,68±4,32	0,551
Contagem de neutrófilos (×10^3^/μL)	7,04±2,45^a^	4,20±2,11^b^	4,13±2,14^b^	**<0,001**
Contagem de linfócitos (×10^3^/μL)	1,21±0,64^a^	1,39±0,94^b^	1,42±0,87^b^	**<0,001**
Monócitos (X10^3^/µl)	1,57±1,44	1,53±1,31	1,56±0,99	0,088
Plaquetas (x10^3^/µl)	339,27±81,13^a^	330,81±77,63^b^	329,64±81,9^b^	**<0,001**
Colesterol LDL (mg/dl)	134,41 ± 37,8	123,6 ± 40,0	121,8±42,74	**<0,001**
Colesterol HDL (mg/dl)	38,79±11,7	41,21±10,4	41,17±9,87	0,107
Triglicerídeos, (mg/dl)	221,8±31,31	187,5 ± 23,6	185,4 ± 22,4	**<0,001**
NT-proBNP (pg/ml)	47,45±11,23^a^	45,2±9,7^b^	43,8±9,3^c^	0,034
Troponina (ng/mL)	98,43±27,46^a^	56,24±24,78^b^	55,37±23,48^b^	**<0,001**
PCR (mg/L)	44,21±8,96^a^	22,41±7,41^b^	21,37±6,99^b^	**<0,001**
**Achados ecocardiográficos**				
FEVE (%)	51,44±2,3	52,34±1,9	53,01±2,1	0,428
IMVE (g/m)	112,13±10,3	112,01±8,6	111,12±4,8	0,559
Tamanho AE (mm)	34,41±3,7	33,52±4,01	33,25±3,9	0,571
**Parâmetros angiográficos**				
Lesão DAE n(%)	220 (41,35)^a^	189 (40,99)^a^	110 (24.01)^b^	**<0,001**
Lesão CX n(%)	200 (37,59)^a^	172(37,31)^a^	90 (19,65)^b^	**<0,001**
Lesão ACD n(%)	155 (29.13)^a^	142 (30,80)^a^	52 (11,35)^b^	**<0,001**
Doença de três vasos n(%)	30 (5,63)^a^	22 (4,77)^b^	6 (1,31)^c^	**<0,001**
**Medicamentos**				
Ácido acetilsalicílico	240 (45.11)^a^	205 (44,46)^a^	102 (22.27)^b^	**<0,001**
IECA, BRA	162 (30,45)	142 (30,80)	140 (30,56)	0,153
Betabloqueador	146 (27,44)	128 (27,76)	120 (26,20)	0,739
Estatina	152 (28,57)^a^	123 (26,68)^a^	102 (22.27)^b^	**0,003**
Clopidogrel	33 (6,20)	27 (5,85)	25 (5,4)	0,224
Bloqueadores dos canais de cálcio	61 (11,46)	52 (11,27)	50 (10,91)	0,786

^abc^Nas explicações sobrescritas, as mesmas letras mostram que não há diferença estatisticamente significativa entre elas, e letras diferentes mostram que há uma diferença estatisticamente significativa. Valores em negrito indicam valores de p estatisticamente significativos (p<0,05). IECA: inibidor da enzima conversora da angiotensina; ALT: alanina Aminotransferase; BRA: bloqueador do Receptor da Angiotensina; AST: aspartato Aminotransferase; IMC: índice de massa corporal; DAC: doença arterial coronária; CAD-RADS: doença arterial coronária - sistema de notificação e dados; PCR: proteína C-reativa; CX: artéria Circunflexa; HDL: lipoproteína de Alta Densidade; AE: átrio esquerdo; DAE: artéria descendente anterior esquerda; LDL: lipoproteína de baixa densidade; FEVE: fração de ejeção do ventrículo esquerdo; IMVE: índice de massa ventricular esquerda; IM: infarto do miocárdio; NT-proBNP: peptídeo natriurético procerebral n-terminal; DAP: doença arterial periférica; ACD: artéria coronária direita; TSH: hormônio estimulante da tireoide.

**Figura 1:**
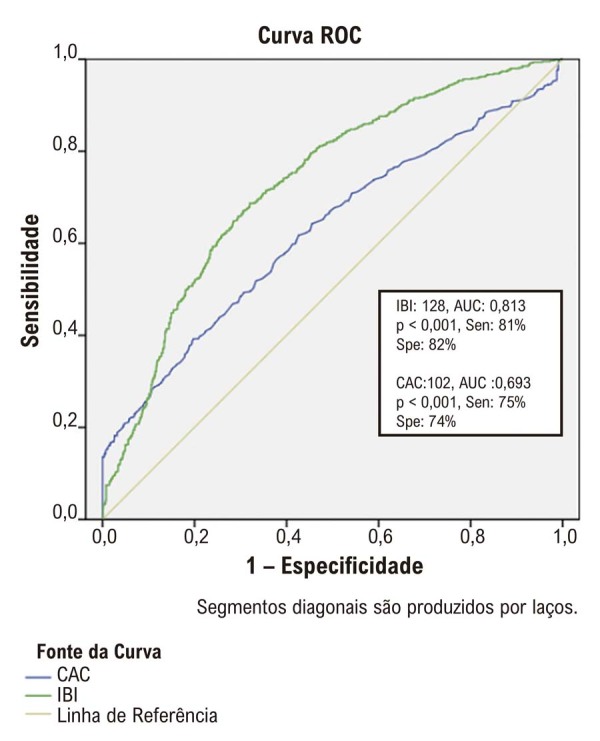
– Curvas ROC dos escores IBI e CAC para previsão de infarto do miocárdio em pacientes com doença arterial coronariana.

**Figura 2:**
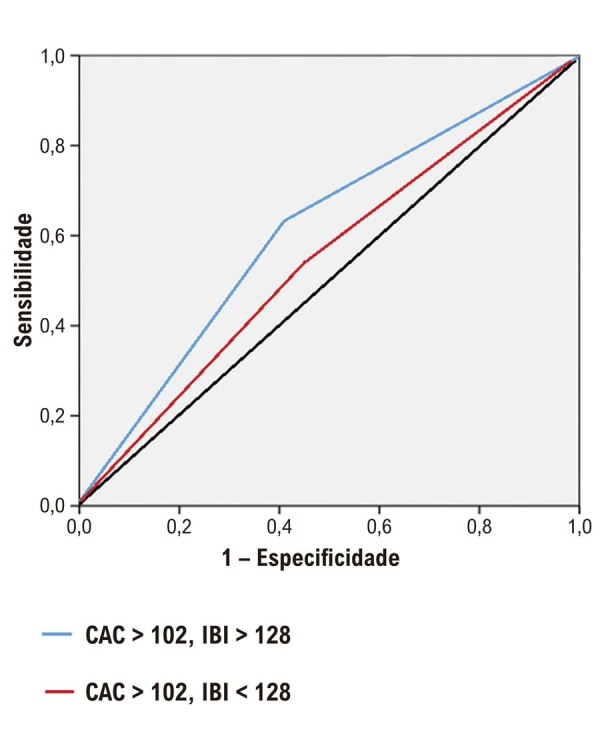
– Análise da curva ROC dos valores de escore de cálcio da artéria coronária (CAC) e índice de carga inflamatória (IBI), e a combinação de CAC e IBI para prever infarto do miocárdio.

A análise ROC determinou os valores de corte ideais para os escores IBI e CAC na predição de IAM ( Figura 1 ). A combinação de ambos os parâmetros demonstrou os maiores níveis de sensibilidade e especificidade na predição de IM em pacientes com escores CAC e IBI elevados ( Figura 2 ).

Na análise de regressão logística multivariada, diabetes mellitus, hiperlipidemia, DAC, contagem de neutrófilos, colesterol LDL, PCR, escore CAC e IBI foram determinados como preditores potenciais independentes de IAM ( Tabela 3 ).

**Tabela 3:** – Análise de regressão univariada e multivariada para identificar preditores independentes em pacientes com infarto do miocárdio

Variável	Análise Univariada		Análise Multivariada	
	OR (IC 95%)	Valor-p	OR (IC 95%)	Valor-p
Hiperlipidemia	1,234 (0,998-1,379)	**0,017**	1,198 (0,745-1,543)	**0,047**
Diabetes mellitus	1,997 (0,982-2,134)	**0,003**	1,887 (0,996-2,245)	**0,032**
DAC	1,392 (1,048-1,592)	**0,040**	1,212 (0,896-1,531)	**0,046**
Uso de estatinas	1,028 (0,883-1,126)	**0,011**	0,952 (0,791-1,113)	0,143
Contagem de neutrófilos (x10^3^/µl)	1,124 (0,950-1,328)	**<0,001**	0,852 (0,657-1,145)	0,053
Contagem de linfócitos (x10^3^/µl)	1,234 (0,997-1,438)	**<0,001**	0,763 (0,443-0,934)	0,159
Plaquetas (x10^3^/µl)	1,107 (0,761-1,278)	**<0,001**	0,972 (0,752-1,129)	0,093
Colesterol LDL (mg/dl)	1,192 (0,837-1,349)	**<0,001**	1,292 (0,823-1,338)	**0,043**
Colesterol HDL (mg/dl)	1,237 (0,973-1,425)	**0,048**	0,937 (0,738-1,173)	0,434
Triglicerídeos, (mg/dl)	1,058 (0,871-1,135)	**0,039**	0,932 (0,759-1,089)	0,537
Troponina (ng/mL)	1,473 (1,102-1,810)	**<0,001**	0,984 (0,749-1,227)	0,052
NT-proBNP (pg/ml)	1,342 (0,988-1,550)	**0,028**	0,892 (0,634-0,913)	0,376
PCR (mg/L)	1,627 (1,204-1,971)	**<0,001**	1,273 (1,104-1,426)	**0,027**
Escore CAC	2,014 (1,283-2,316)	**<0,001**	1.930 (1.254-2.364)	**0,018**
CAD-RADS	1,539 (1,011-1,679)	**<0,001**	1.430 (1.112-1.619)	**0,035**
IBI	2.234 (1.376-2.891)	**<0,001**	2.129 (1.637-2.461)	**<0,001**

Valores em negrito indicam valores de p estatisticamente significativos (p<0,05). CAC: cálcio da artéria coronária; DAC: doença arterial coronária; CAD-RADS: Sistema de notificação e dados sobre doenças arteriais coronárias; PCR: proteína C-reativa; HDL: lipoproteína de alta densidade; IBI: índice de carga inflamatória; LDL: lipoproteína de baixa densidade; NT-proBNP: peptídeo natriurético tipo B N-terminal.

**Figura 3:**
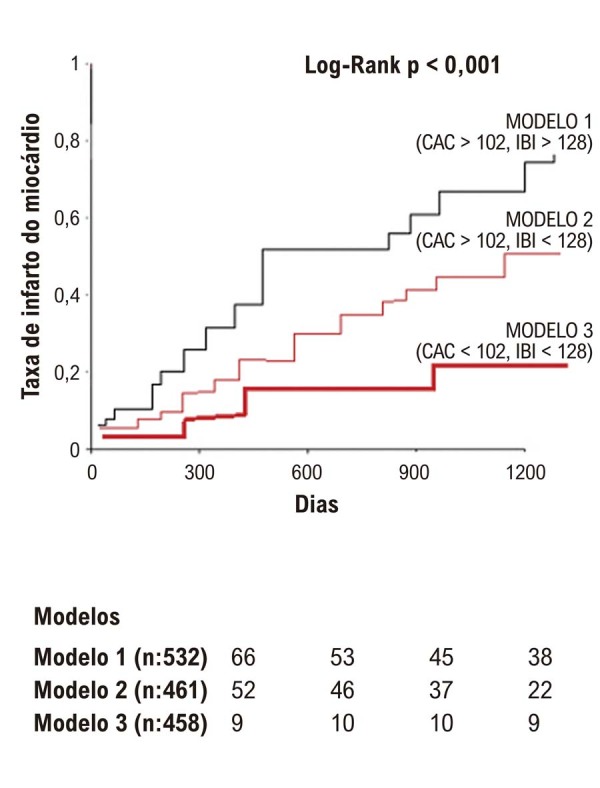
– Taxa de infarto do miocárdio de acordo com modelos.

Em seguida, avaliamos se os escores IBI e CAC estavam incrementalmente associados ao risco de IAM em nossa coorte de acompanhamento de doença arterial coronariana de longo prazo. Curvas de Kaplan-Meier foram usadas para analisar as diferenças entre modelos com diferentes questões de IBI e CAC na predição de MACEs ( Figura 3 ). Pacientes com escores IBI e CAC mais altos eram mais propensos a ter IM do que aqueles com escores IBI e CAC mais baixos (log-rank p < 0,001). De acordo com nosso sistema de modelagem, uma taxa de IM significativamente maior foi encontrada no grupo Modelo 1 do que no grupo Modelo 2 e no grupo Modelo 2 do que no grupo Modelo 3 nesta coorte de acompanhamento de longo prazo. Após ajuste de risco, 397 (32,14%) pacientes desenvolveram IAM durante o acompanhamento ( Figura 3 ). Pacientes com CAC alto (> 102) e IBI (> 128) estavam em maior risco de IAM (P < 0,001 para todos). Após o ajuste de risco, 202 pacientes (37,9%) apresentaram um IAM em 3 anos no grupo de pacientes do Modelo 1 (razão de chances [OR] 1,484 [1,287-1,750]), 157 pacientes (34,05%) apresentaram um IAM em 3 anos no grupo de pacientes do Modelo 2 ([OR] 1,184 [0,867-1,340]) e 38 pacientes (8,29%) apresentaram um IAM em 3 anos no grupo de pacientes do Modelo 3 ([OR] 0,384 [0,237-0,431]).

## Discussão

Neste estudo retrospectivo, investigamos o valor preditivo dos escores IBI e CAC na identificação de pacientes com maior risco de IM (placa vulnerável) em pacientes com placa submetidos à angiotomografia por dor torácica. Nossos achados sugerem que tanto o IBI quanto o CAC são preditores significativos de IAM para placa vulnerável, com escores mais altos associados a risco aumentado. Esses resultados reforçam a potencial utilidade desses índices na prática clínica para a identificação precoce de pacientes de alto risco e podem auxiliar no refinamento de estratégias terapêuticas.

Os níveis elevados de neutrófilos, PCR, colesterol LDL, triglicerídeos, NT-proBNP e troponina observados no grupo com IAM, em comparação com o grupo sem IAM, são consistentes com o papel bem estabelecido da inflamação e do metabolismo lipídico na patogênese da aterosclerose e subsequente ruptura da placa.^13 , 14^ Os valores significativamente mais elevados de IBI no grupo com IAM reforçam a hipótese de que a inflamação sistêmica contribui para o desenvolvimento de síndromes coronarianas agudas (SCA).^15^ Esse achado está alinhado com pesquisas anteriores que demonstram que biomarcadores inflamatórios são poderosos preditores de eventos cardiovasculares.^16^

Da mesma forma, os escores CAC mais altos no grupo IAM em comparação ao grupo sem IAM confirmam o papel da calcificação da artéria coronária como um marcador da carga da placa aterosclerótica.^17^ A associação entre escores CAC mais altos e um risco aumentado de IAM foi amplamente documentada na literatura, onde é frequentemente usada para estratificar pacientes em diferentes categorias de risco para eventos cardiovasculares futuros.^18^ A observação de que os pacientes no Modelo 1 (escores IBI e CAC altos) apresentaram as maiores taxas de IAM, seguidos por aqueles no Modelo 2 e, em seguida, no Modelo 3, reforça ainda mais o uso combinado desses índices para estratificação de risco.^19^

A aterosclerose coronária se manifesta com o aparecimento de estrias de gordura nas artérias coronárias e outros leitos arteriais nos estágios iniciais da vida. De fato, se sabe hoje que a aterosclerose começa durante o desenvolvimento fetal, especialmente em fetos de mães com hipercolesterolemia.^20^ No desenvolvimento da aterosclerose, primeiro ocorre disfunção endotelial, depois acúmulo de lipídios e macrófagos leucocitários na camada íntima, depois inflamação e, finalmente, proliferação e migração de células musculares lisas da camada média, resultando na formação de uma capa fibrosa na lesão gordurosa. Essa lesão é agora uma lesão complexa irreversível.^21^ Desde a publicação da classificação CAD-RADS original, vários estudos prospectivos forneceram evidências que apoiam a utilidade clínica da angiotomografia coronária e a importância dos achados tomográficos em pacientes com suspeita de doença arterial coronária estável. Estes incluem os estudos PROMISE^22^ e SCOT-HEART,^23^ que demonstraram que a ATC é clinicamente útil como alternativa aos testes funcionais ou como adjuvante ao tratamento padrão. Com base nesses estudos e em múltiplos registros, o valor prognóstico da classificação CAD-RADS foi validado, com escores CAD-RADS mais altos sendo associados a riscos aumentados de IM fatal e não fatal.^9 , 24^ Nosso estudo corrobora esses estudos e constatou que pacientes submetidos à CAD-RADS apresentaram escores mais altos.

A análise ROC forneceu evidências adicionais que corroboram a utilidade dos escores IBI e CAC como preditores de IAM. Os valores de corte identificados para IBI (128) e CAC (102) demonstraram boa sensibilidade e especificidade, sugerindo que esses índices podem servir como marcadores confiáveis para avaliar o risco de IM em pacientes com dor torácica. Além disso, a análise de regressão logística multivariada identificou diabetes, hiperlipidemia, doença arterial coronariana (DAC), contagem de neutrófilos, colesterol LDL, PCR, escore CAC e IBI como preditores independentes de IAM, destacando a natureza multifatorial dessa condição.^25^

Um dos achados mais significativos do nosso estudo é o valor incremental da combinação dos escores IBI e CAC na previsão de eventos cardiovasculares adversos maiores (MACEs) a longo prazo. A análise de Kaplan-Meier mostrou um gradiente claro no risco de IAM entre os três modelos, com o maior risco em pacientes com altos escores IBI e CAC. Isso sugere que a integração dos índices inflamatórios e de calcificação pode fornecer uma avaliação mais abrangente do risco cardiovascular do paciente, potencialmente orientando intervenções terapêuticas mais personalizadas.^3 , 26^

A combinação de marcadores sistêmicos como CAC e IBI nos permite identificar não apenas placas vulneráveis individuais, mas também “pacientes vulneráveis” que podem estar em maior risco para eventos cardiovasculares futuros. O conceito de paciente vulnerável representa uma abordagem mais holística que abrange a carga aterosclerótica sistêmica, o estado inflamatório e a vulnerabilidade miocárdica, em vez de focar em uma única característica da placa. Em um paciente com altos escores de CAC e IBI que possui múltiplas placas não obstrutivas, o fato de uma placa levar a IAM e outra não é provavelmente o resultado de interações complexas entre fatores locais (função endotelial, morfologia da placa) e fatores sistêmicos (inflamação, tendência à coagulação). Portanto, o uso de CAC e IBI ajuda a definir o paciente vulnerável, em vez de apenas placas vulneráveis específicas.

Nosso estudo apresenta diversas limitações que devem ser consideradas na interpretação dos resultados. Primeiro, o desenho retrospectivo pode introduzir viés de seleção, e a exclusão de pacientes com dados incompletos pode ter influenciado nossos achados. Segundo, embora a população do estudo tenha sido relativamente grande, ela foi proveniente de um único centro, o que pode limitar a generalização dos resultados. Estudos futuros devem ter como objetivo validar esses achados em coortes maiores e multicêntricas e explorar os potenciais benefícios de terapias direcionadas com base nos escores IBI e CAC.

Apesar das limitações do nosso delineamento retrospectivo unicêntrico, esses achados sugerem que a integração dos índices inflamatórios e de calcificação oferece uma avaliação mais abrangente do risco cardiovascular. Mais pesquisas prospectivas multicêntricas são necessárias para confirmar esses achados e explorar como esses índices podem ser integrados à prática clínica de rotina para melhorar os desfechos dos pacientes.

## Conclusão

Em conclusão, nosso estudo corrobora o uso dos escores IBI e CAC como ferramentas valiosas para a predição de IAM em pacientes submetidos à angiotomografia. Identificamos valores de corte ótimos de 128 para IBI e 102 para o escore CAC, o que demonstrou boa sensibilidade e especificidade na predição de eventos futuros de IAM. A combinação desses índices parece melhorar a estratificação de risco, com pacientes com IBI e CAC elevados apresentando risco significativamente maior de IAM durante nosso período de acompanhamento de 3 anos.

Nosso sistema de três modelos fornece aos médicos uma estrutura prática para identificar pacientes de alto risco que podem se beneficiar de monitoramento mais intensivo e intervenções preventivas. Essa abordagem nos permite identificar não apenas placas vulneráveis, mas também “pacientes vulneráveis”, que são mais suscetíveis a eventos cardiovasculares na presença de aterosclerose sistêmica e inflamação, proporcionando assim uma perspectiva mais holística na avaliação de risco. Essa abordagem pode potencialmente permitir estratégias terapêuticas mais personalizadas em pacientes de alto risco, particularmente aqueles com evidências de calcificação e inflamação sistêmica.

### Destaques

1. A combinação dos escores IBI e CAC proporciona melhor estratificação de risco para a previsão de futuro IM (placa vulnerável) em pacientes com placa detectada por angiografia por TC.
2. Nosso estudo é o primeiro a usar o escore CAC e o IBI juntos.
3. O conceito de “paciente vulnerável”, incorporando as escores CAC e IBI, fornece uma abordagem mais abrangente à avaliação do risco cardiovascular do que focar apenas nas características individuais da placa.

## References

[1] Schuijf JD, Pundziute G, Jukema JW, Lamb HJ, van der Hoeven BL, de Roos A (2006). Diagnostic Accuracy of 64-Slice Multislice Computed Tomography in the Noninvasive Evaluation of Significant Coronary Artery Disease. Am J Cardiol.

[2] Libby P (2002). Inflammation in Atherosclerosis. Nature.

[3] Naghavi M, Libby P, Falk E, Casscells SW, Litovsky S, Rumberger J (2003). From Vulnerable Plaque to Vulnerable Patient: A Call for New Definitions and Risk Assessment Strategies: Part I. Circulation.

[4] Ridker PM, Rifai N, Rose L, Buring JE, Cook NR (2002). Comparison of C-Reactive Protein and Low-Density Lipoprotein Cholesterol Levels in the Prediction of First Cardiovascular Events. N Engl J Med.

[5] Xie H, Ruan G, Wei L, Deng L, Zhang Q, Ge Y (2023). The Inflammatory Burden Index is a Superior Systemic Inflammation Biomarker for the Prognosis of Non-Small Cell Lung Cancer. J Cachexia Sarcopenia Muscle.

[6] Xie H, Ruan G, Ge Y, Zhang Q, Zhang H, Lin S (2022). Inflammatory Burden as a Prognostic Biomarker for Cancer. Clin Nutr.

[7] Yu F, Peng J (2024). Association between Inflammatory Burden Index and Cardiovascular Disease in Adult Americans: Evidence from NHANES 2005-2010. Heliyon.

[8] Du M, Xu L, Zhang X, Huang X, Cao H, Qiu F (2023). Association between Inflammatory Burden Index and Unfavorable Prognosis after Endovascular Thrombectomy in Acute Ischemic Stroke. J Inflamm Res.

[9] Xie JX, Cury RC, Leipsic J, Crim MT, Berman DS, Gransar H (2018). The Coronary Artery Disease-Reporting and Data System (CAD-RADS): Prognostic and Clinical Implications Associated with Standardized Coronary Computed Tomography Angiography Reporting. JACC Cardiovasc Imaging.

[10] Williams MC, Moss A, Dweck M, Hunter A, Pawade T, Adamson PD (2020). Standardized Reporting Systems for Computed Tomography Coronary Angiography and Calcium Scoring: A Real-World Validation of CAD-RADS and CAC-DRS in Patients with Stable Chest Pain. J Cardiovasc Comput Tomogr.

[11] Budoff MJ, Shaw LJ, Liu ST, Weinstein SR, Mosler TP, Tseng PH (2007). Long-Term Prognosis Associated with Coronary Calcification: Observations from a Registry of 25,253 Patients. J Am Coll Cardiol.

[12] Arbab-Zadeh A, Fuster V (2015). The Myth of the "Vulnerable Plaque": Transitioning from a Focus on Individual Lesions to Atherosclerotic Disease Burden for Coronary Artery Disease Risk Assessment. J Am Coll Cardiol.

[13] Byrne RA, Rossello X, Coughlan JJ, Barbato E, Berry C, Chieffo A (2023). 2023 ESC Guidelines for the Management of Acute Coronary Syndromes. Eur Heart J.

[14] Libby P, Ridker PM, Hansson GK (2011). Progress and Challenges in Translating the Biology of Atherosclerosis. Nature.

[15] Mangalesh S, Dudani S, Mahesh NK (2024). Development of a Novel Inflammatory Index to Predict Coronary Artery Disease Severity in Patients with Acute Coronary Syndrome. Angiology.

[16] Hansson GK, Hermansson A (2011). The Immune System in Atherosclerosis. Nat Immunol.

[17] Budoff MJ, Hokanson JE, Nasir K, Shaw LJ, Kinney GL, Chow D (2010). Progression of Coronary Artery Calcium Predicts All-Cause Mortality. JACC Cardiovasc Imaging.

[18] Greenland P, Blaha MJ, Budoff MJ, Erbel R, Watson KE (2018). Coronary Calcium Score and Cardiovascular Risk. J Am Coll Cardiol.

[19] Schuijf JD, Shaw LJ, Wijns W, Lamb HJ, Poldermans D, de Roos A (2005). Cardiac Imaging in Coronary Artery Disease: Differing Modalities. Heart.

[20] Napoli C, Glass CK, Witztum JL, Deutsch R, D'Armiento FP, Palinski W (1999). Influence of Maternal Hypercholesterolaemia during Pregnancy on Progression of Early Atherosclerotic Lesions in Childhood: Fate of Early Lesions in Children (FELIC) Study. Lancet.

[21] Williams KJ, Tabas I (1998). The Response-to-Retention Hypothesis of Atherogenesis Reinforced. Curr Opin Lipidol.

[22] Douglas PS, Hoffmann U, Patel MR, Mark DB, Al-Khalidi HR, Cavanaugh B (2015). Outcomes of Anatomical versus Functional Testing for Coronary Artery Disease. N Engl J Med.

[23] SCOT-HEART investigators (2015). CT Coronary Angiography in Patients with Suspected Angina Due to Coronary Heart Disease (SCOT-HEART): An Open-Label, Parallel-Group, Multicentre Trial. Lancet.

[24] Nam K, Hur J, Han K, Im DJ, Suh YJ, Hong YJ (2019). Prognostic Value of Coronary Artery Disease-Reporting and Data System (CAD-RADS) Score for Cardiovascular Events in Ischemic Stroke. Atherosclerosis.

[25] Mozaffarian D, Benjamin EJ, Go AS, Arnett DK, Blaha MJ, Cushman M (2016). Heart Disease and Stroke Statistics-2016 Update: A Report from the American Heart Association. Circulation.

[26] Naghavi M, Libby P, Falk E, Casscells SW, Litovsky S, Rumberger J (2003). From Vulnerable Plaque to Vulnerable Patient: A Call for New Definitions and Risk Assessment Strategies: Part II. Circulation.

