# Supplementary material for: Placas Vulneráveis Detectadas por TC Cardíaca e Risco de Infarto Agudo do Miocárdio Futuro: Valor Preditivo dos Escores CAC e do Índice de Carga Inflamatória
Source: Arq Bras Cardiol. 2025 Dec 8;122(11):e20250052. [Article in Portuguese] doi: 10.36660/abc.20250052 (PMC12711219; doi:10.36660/abc.20250052)
Supplement: Translation

# Translation: Cardiac CT-Detected Vulnerable Plaques and Risk of Future Acute Myocardial Infarction: Predictive Value of CAC Scores and Inflammatory Burden Index

**DOI:** 10.36660/abc.20250052i

**Authors:** Sefa Erdi Ömür, Emin Koyun, Gökhan Cabri

**Affiliations:** 1 Türkiye Department of Cardiology, Tokat Gaziosmanpaşa University, Tokat – Türkiye; 2 Türkiye Cardiology Clinic, Sivas State Hospital, Sivas – Türkiye

**Keywords:** Coronary Vessels, Myocardial Infarction, Computed Tomography Angiography

## Abstract

**Background:**

Computed Tomography (CT) angiography is widely used as a basic non-invasive modality to evaluate coronary artery disease. Inflammatory burden index (IBI) is a systemic inflammation indicator reflecting the inflammatory status. It is still difficult to determine the high risk of future acute myocardial infarction (MI) in patients with plaque detected by CT angiography.

**Objective:**

This study aimed to evaluate the effectiveness of CAC scores and IBI in predicting MI risk among patients undergoing CT angiography for chest pain. We sought to assess the individual and combined predictive value of these indices, determine their optimal cut-off values, and compare MI risk across patient groups with varying CAC and IBI scores. We also aimed to evaluate the potential role of these markers in identifying ‘vulnerable patients’ at high risk for future cardiovascular events.

**Methods:**

This retrospective study included 1235 patients who underwent CT angiography due to chest pain and were followed for 3 years (2.8-3.4). Patients were categorized into three models based on their CAC and IBI scores. The primary outcome was the occurrence of MI during the follow-up period. Statistical significance was established at p < 0.05 for all analyses.

**Results:**

Patients who experienced MI had significantly higher IBI and CAC scores compared to those without MI. Logistic regression analysis identified CAC score and IBI as independent predictors of MI. ROC analysis determined optimal cut-off values for IBI (128) and CAC (102) in predicting MI. Kaplan-Meier analysis revealed a significant gradient in MI risk across the three models, with the highest risk observed in patients with both high IBI and CAC scores.

**Conclusion:**

The combination of IBI and CAC scores provides improved risk stratification for the prediction of future MI (vulnerable plaque) in patients with plaque detected by CT angiography. This approach may facilitate more specific treatment strategies for high-risk patients.

## Introduction

Acute myocardial infarction (AMI) remains one of the leading causes of morbidity and mortality worldwide. Despite advances in diagnostic techniques and therapeutic interventions, the early identification and prediction of MI, particularly in patients presenting with chest pain, continue to pose significant challenges for clinicians. Computed tomography (CT) angiography has emerged as a crucial tool in the non-invasive evaluation of coronary artery disease (CAD) and plaque characterization, providing valuable insights into the presence and extent of coronary atherosclerosis, as well as the identification of vulnerable plaques prone to rupture.^1^

Inflammation plays a pivotal role in the pathogenesis of atherosclerosis and plaque destabilization, leading to MI.^2^Recent years have witnessed a paradigm shift in cardiovascular medicine from focusing solely on vulnerable plaques toward a more comprehensive vulnerable patient concept. The vulnerable patient encompasses not only local plaque characteristics but also factors such as systemic inflammation and widespread atherosclerotic burden.^3^Our study explores how the combination of systemic markers like Coronary Artery Calcium Score (CAC) and Inflammatory Burden Index (IBI) may help identify not just vulnerable plaques but also vulnerable patients susceptible to future cardiovascular events. Biomarkers of inflammation, such as C-reactive protein (CRP) and the neutrophil-to-lymphocyte ratio (NLR), have been recognized as valuable predictors of cardiovascular events.^4^Recent studies have examined the relationship between inflammation burden and potential cardiovascular disease. Researchers combined CRP and NLR to create the IBI measure, which is now widely used in cancer and cerebrovascular disease prognosis studies.^5 , 6^A recent study found an association between IBI levels and the prevalence of cardiovascular disease.^7^Compared with CRP and NLR, IBI, a comprehensive marker of inflammation, can provide a more consistent assessment of inflammation, more accurately reflect the inflammatory status of the body, better assess the effectiveness of treatment, and predict the prognosis of patients.^8^The Coronary Artery Disease-Reporting and Data System (CAD-RADS) has been shown to accurately predict major adverse cardiovascular events, defined as unstable angina, myocardial infarction (MI), or death, in patients with stable chest pain, with superior performance compared with traditional risk factors, other risk stratification scores, the CAC, and the previous society of cardiovascular computed tomography coronary stenosis scoring system.^9 , 10^

Moreover, the CAC score, a well-established measure of atherosclerotic plaque burden, has been extensively used to stratify risk in patients with CAD.^11^However, the relative contribution of CAC and IBI scores in predicting plaque erosion, which is a precursor to MI, remains to be fully elucidated. Understanding the relationship between these indices and their ability to predict plaque instability could significantly impact clinical decision-making and patient management.^12^

This study aims to evaluate the effectiveness of CAC and IBI scores in predicting plaque erosion and subsequent MI in patients who present with chest pain and undergo CT angiography. By examining these factors retrospectively in a large cohort, this study seeks to provide insights into their utility in improving risk stratification and guiding therapeutic interventions in the outpatient cardiology setting.

## Methods

### Study population and data collection

This prospective cohort investigation was conducted at the cardiology clinic of a local university hospital. The study population consisted of patients who presented with stable angina pectoris or atypical chest pain and underwent diagnostic CT angiography between June 2019 and June 2021. The study included patients who did not have unstable angina at baseline. During the follow-up period, some patients developed MI while others remained event-free. All patients were monitored for MI events throughout the study period. Of the 1235 patients included in the final analysis, 283 patients experienced MI during follow-up, while 952 patients remained free of MI events. The patients included in the study had initial troponin values within the normal range and no typical changes on their electrocardiograms suggestive of MI infarction. Medical treatments (acetylsalicylic acid, antihyperlipidemic therapy, etc.) of the patients were optimized after CT angiography. All patients included in the study were then prospectively followed for MI events until June 2024, resulting in a mean follow-up of 3 (2.8-3.4) years.

CT angiographic images were analyzed to identify plaques, with specific focus on lesions demonstrating less than 50% stenosis. Among patients who experienced MI during the follow-up period, their baseline CT angiograms were retrospectively evaluated to characterize the culprit plaques. Vulnerable plaque characteristics were systematically evaluated using established computed tomography imaging criteria. The assessment included quantitative measurement of plaque attenuation, with low-attenuation plaques defined as those with <30 Hounsfield units. Positive remodeling was identified by a remodeling index exceeding 1.1, calculated as the ratio of the vessel diameter at the plaque site to the reference diameter of the normal proximal segment. Additional high-risk plaque features that were evaluated included spotty calcification patterns and the presence of napkin-ring sign (NRS). These validated CT imaging markers were analyzed in conjunction with CAC scores and IBI to provide a comprehensive assessment of plaque vulnerability. All CT images were independently evaluated by two experienced cardiovascular radiologists who were blinded to the clinical outcomes, with any discrepancies resolved by consensus.

Initially, 1436 patients met the inclusion criteria; however, 201 were excluded due to incomplete data or declined participation ( Central Illustration ), resulting in a final study cohort of 1235 patients. Demographic, clinical, medical, and angiographic characteristics of the patients and 3-year follow-up results were obtained using hospital patient data and hospital database. The study complies with the principles stated in the Declaration of Helsinki and was approved by the local university hospital ethics committee. The entirety of this manuscript was written without the use of any artificial intelligence (AI)-assisted technologies, including large language models, chatbots, or image creators. All content was produced solely by the authors using traditional writing and research methods.

Patient outcomes were systematically monitored through a comprehensive, multi-modal follow-up protocol. All enrolled patients were scheduled for regular clinical visits at our institution, where detailed cardiovascular assessments were performed and documented. For patients who missed scheduled appointments, structured telephone interviews were conducted with either the patients or their designated next of kin using contact information from our hospital database. In cases where patients reported MI events at other healthcare facilities, our research team established direct communication with the treating cardiologists at those institutions. Additionally, all patient events were verified through the national electronic health records system (e-Nabiz), which provided access to angiographic images and comprehensive clinical data from any healthcare facility within the national network. This multi-faceted approach to patient monitoring, combining direct clinical follow-up, systematic telecommunication, inter-institution physician consultation, and centralized electronic health record review, enabled complete documentation and verification of MI events regardless of their occurrence location.

The European Society of Cardiology Guideline criteria were used to diagnose MI in patients^13^. Patients who had MI at the indicated plaque point according to the CT angiography result (n: 283) and those who did not (n: 952) were included in the study, and the patients were evaluated in terms of IBI and CAC scores. Three modeling systems were created to evaluate the success of IBI and CAC scores in predicting plaque erosion in patients. According to this modeling system, patients with high CAC and IBI scores were included in the patient group in model 1. Patients with high CAC scores and low IBI values were included in the model 2 group. Patients with low IBI and CAC scores were included in the model 3 group. CAC and IBI values were added to the models as continuous variables according to the cut-off value obtained from the ROC analysis. Patients were followed for an average of 3 (2.8-3.4) years according to three models, and MI rates were recorded. The CAD-RADS (Coronary Artery Disease-Reporting and Data System) score, which was developed to standardize coronary CT imaging reporting, improve communication, and guide treatment, was evaluated and recorded for patients. The inflammation burden index is defined as the product of CRP multiplied by NLR. Patients with any systemic inflammatory and rheumatological disease, storage disease, anemia, malignancy, age under 18, acute or chronic stroke, any hematological disease including advanced renal and/or hepatic failure, patients with a history of acute or chronic infection, patients with a history of blood transfusion within the last 3 months, patients with severe valve disease and patients who had valve surgery were excluded from the study. At the same time, patients with stenosis of more than 50% on CT angiography were not included in the study because they were sent directly for angiography.

### Laboratory and demographic examination

All blood samples were obtained from peripheral venous blood immediately before CT angiography. NT-proBNP, CRP, lipid panel, fasting plasma glucose, creatinine, troponin-I, and other routine parameters were obtained from the blood samples. Complete blood count (CBC) was evaluated with an automatic blood cell counter (Coulter LH 780 Hematology Analyzer, Beckman Coulter Corp, Hialeah, Florida, USA). Patients with fasting plasma glucose level > 125 mg/dL, HgA1c level > 6.5% or using antidiabetic drugs (oral/insulin) were considered as DM patients. Patients with low-density lipoprotein cholesterol (LDL-C) levels above 100 mg/dL or using antilipidemic drugs were considered hyperlipidemic (HL) patients. Use of anti-hypertensive drugs or systolic and diastolic blood pressures above 140–90 mmHg was considered as hypertension (HT). CAD was defined as significant stenosis of the coronary arteries, a narrowing of the mean lumen diameter of more than 50%, or evidence of previous myocardial incarnation. In our assessment of MI patients, we considered not only individual plaque characteristics but also systemic factors that might contribute to the overall concept of the ‘vulnerable patient’, individuals at elevated risk for cardiovascular events due to a combination of plaque vulnerability, inflammatory status, and other systemic factors. Patients who had smoked for the last six months were considered smokers.

### Coronary angiography, echocardiographic evaluation

Before coronary angiography, all patients underwent transthoracic echocardiography (TTE) with Vivid E7 (GE Vingmed Ultrasound) echocardiography device and MS5 (1.5-4.5 MHz) ultrasound probe in the left decubitus position. Left ventricular ejection fraction (LVEF) was measured by two experienced cardiologists using the Simpson method. All CAG and PCI procedures were performed with Xper Allura FD-10 Model C Arm Detector System Angiography Device (Philips Medical Systems International B.V., Best, Netherlands). All patients underwent femoral or radial intervention with the standard Judkins technique and a 6 Fr catheter. Two experienced interventional cardiologists performed PCI procedures.

### Statistical analysis

Statistical analyses were performed using SPSS 26.0 software (SPSS Inc., Chicago, IL, USA). The normality of data distribution was assessed using the Kolmogorov-Smirnov test. Categorical variables were expressed as numbers and percentages (n, %), while continuous variables were presented as mean ± standard deviation (mean ± SD) or median with interquartile range (median, IQR) according to their distribution pattern. For parametric data meeting required assumptions (interval/ratio scale measurements with normal distribution), an independent samples t-test was used for comparing two independent groups, while one-way ANOVA was employed for comparing more than two independent groups. When using ANOVA for multiple group comparisons, post-hoc analysis was conducted using Tukey’s test for homogeneous variances and Games-Howell test for non-homogeneous variances to determine which groups differed significantly from others. When parametric assumptions were not met, the Mann-Whitney U test was used for comparing two independent groups. Chi-square test was utilized for analyzing categorical variables. (ROC) Curve analysis was used to calculate the best cut-off values of CAC and IBI. The best cut-off value obtained from the ROC curve of CAC and IBI was taken as the cut-off value in categorizing IBI and CAC score values as high and low. Univariate logistic regression analysis was performed to determine the predictors of MI infarction in all patients. Variables that were significant in univariate logistic regression analysis (p<0.05) were included in multivariate logistic regression analysis. The results of logistic regression analysis are presented as odds ratios (OR) and 95% confidence intervals (CI). The Kaplan-Meier survival curve was used to examine the difference in MI incidence rates between groups, and statistical significance was determined using the Log-rank test. A two-sided p <0.05 was considered statistically significant.

## Results

A total of 1235 patients who underwent CT angiography was included in our study. The basic demographic, clinical, and laboratory characteristics, angiographic results, and medications of the patients included in the study are given in Table 1 . Coronary artery disease, diabetes, and hyperlipidemia were found to be higher in the group with MI. Neutrophil count, platelet, LDL cholesterol, triglyceride, CRP, and NT-proBNP values were significantly higher in the MI group than in the non-MI group. Troponin levels were higher in the MI group as expected. The lymphocyte count was higher in the group without MI. When the IBI value was examined, it was found to be significantly higher in the group with MI than in the group without MI. CAC, statin use, and e/e’ were higher in the MI group compared to the non-MI group.

**Table 1:** – Comparison of demographics, laboratory, and drug medications of the groups

Variable	Myocardial infarction (n: 397)	No myocardial infarction (n: 952)	p
Age (years)	65.41±3.24	64.45±4.03	0.180
Female gender n (%)	174 (43.82)	423 (44.43)	0.639
BMI kg/m^2^	27.43±3.22	26.91±3.14	0.221
Diabetes mellitus n (%)	138 (34.76)	298 (31.30)	**0.003**
Hypertension n (%)	131 (32.99)	321 (33.71)	0.861
Hyperlipidemia n (%)	135 (34.00)	301 (31.61)	**0.017**
Smoking n (%)	140 (35.26)	326 (34.24)	0.283
Atrial fibrillation n (%)	47 (11.83)	111 (11.65)	0.569
CAD n (%)	127 (31.98)	298 (31.30)	**0.040**
PAD n (%)	23 (5.79)	52 (5.46)	0.079
CAD-RADS	2.2±0.53	1.6±0.21	**<0.001**
**Hematological variables**			
Fasting Glucose(mg/dl)	118.62±12.79	116.63±11.78	0.829
Creatinine (mg/dl)	1.01±0.41	0.99±0.55	0.129
Sodium (mmol/L)	138.55±3.76	139.66±2.86	0.873
Potassium (mmol/L)	4.50±0.32	4.51±0.34	0.499
ALT (U/L)	25.39±2.81	24.59±2.87	0.553
AST (U/L)	24.37±3.16	24.51±4.82	0.359
TSH(µIU/ml)	1.44±0.32	1.41±0.29	0.283
Hemoglobin (g/dl)	10.61±3.41	10.53±3.59	0.573
WBC count (x10^3^/µl)	10.11±3.11	9.28±4.07	0.449
Neutrophil count (×10^3^/μL)	8.14±2.44	3.89±2.31	**<0.001**
Lymp. count (×10^3^/μL)	1.11±0.74	1.29±1.94	**<0.001**
Monocyte (X10^3^/µl)	1.85±1.51	1.84±1.31	0.113
Platelet (x10^3^/µl)	349.21±61.23	328.91±66.73	**<0.001**
LDL-cholesterol (mg/dl)	136.44 ± 30.8	125.6 ± 41.12	**<0.001**
HDL-cholesterol(mg/dl)	37.69±12.41	42.26±10.1	**0.048**
Triglyceride, (mg/dl)	231.81±32.21	218.51 ± 27.6	**0.039**
NT-proBNP (pg/ml)	52.41±12.32	44.2±10.7	**0.028**
Troponin (ng/mL)	99.53±29.36	57.24±25.68	**<0.001**
CRP (mg/L)	45.24±9.56	21.42±8.41	**<0.001**
IBI	326.88±32.57	64.49±15.21	**<0.001**
**Echocardiographic Findings**			
LVEF (%)	52.41±2.6	52.51±2.9	0.947
LA size (mm)	35.51±4.8	34.42±5.01	0.557
LVMI (g/m)	112.32±2.3	111.2±3.6	0.469
**Angiographic parameters**			
LAD lesion n (%)	166 (41.81)	398 (41.80)	0.783
CX lesion n (%)	138 (34.76)	342 (35.92)	0.663
RCA lesion n (%)	120 (30.22)	296 (31.09)	0.239
Three vessel disease n (%)	20 (5.03)	51 (5.35)	0.570
CAC score	342±25.31	88±12.3	**<0.001**
**Medications**			
Acetylsalicylic acid	178 (44.83)	425 (44.62)	0.601
ACE I, ARB	123 (30.98)	288 (30.25)	0.122
Beta blocker	91 (22.92)	220 (23.10)	0.806
Statin	111 (27.95)	218 (22.89)	**0.011**
Clopidogrel	16 (4.03)	43 (4.51)	0.347
Calcium channel blockers	39 (9.82)	99 (10.39)	0.779

Bold values indicate statistically significant p values (p<0.05). ACE I: Angiotensin Converting Enzyme Inhibitor; ALT: alanine aminotransferase; ARB: angiotensin receptor blocker; AST: aspartate Aminotransferase; BMI: body mass index; CAC: coronary artery calcium; CAD: coronary artery disease; CAD-RADS: coronary artery disease-reporting and data system; CRP: C-Reactive protein; CX: circumflex artery; HDL: high-density lipoprotein; IBI: inflammatory burden index; LA:,lLeft atrium; LAD: left anterior descending artery; LDL: low-density lipoprotein; LVEF: left ventricular ejection fraction; LVMI: left ventricular mass index; NT-proBNP: N-terminal pro-brain natriuretic peptide; PAD: peripheral artery disease; RCA: right coronary artery; TSH: thyroid stimulating hormone; WBC: white blood cell.

Five hundred and thirty-two patients (36.66%) were included in the patient group in model 1, 461 patients (31.77%) were included in the patient group in model 2, and 458 patients (31.56%) were included in the patient group in model 3. Classification of patients according to IBI and CAC scores is shown in Table 2 . Coronary artery disease, diabetes, and hyperlipidemia were found to be higher in the Model 1 group compared to the other two groups. Neutrophil count, platelet, CRP, LDL cholesterol, triglyceride, NT-proBNP, and troponin were found to be higher in the Model 1 group compared to the other two groups. In the angiographic evaluation, LAD, LCX, and RCA lesion rates were similar in the Model 1 and Model 2 groups, while they were found to be significantly higher in these two groups compared to the Model 3 group. In the echocardiographic evaluation, e/e’ was found to be higher in the Model 1 group compared to the other two groups. The use of acetylsalicylic acid and statins in drug medication was found to be lower in the Model 3 group compared to the other two groups.

**Table 2:** – Comparison of demographics, laboratory, and drug medications of the models

Variable	MODEL 1 (n: 532)	MODEL 2 (n: 461)	MODEL 3 (n: 458)	p
Age (years)	65.21±1.14	65.25±3.03	64.79±8.68	0.263
Female gender n (%)	248 (46.61)	210 (45.55)	209 (45.63)	0.429
BMI kg/m^2^	27.23±4.22	28.01±3.84	28.03±4.74	0.367
Diabetes mellitus n (%)	172 (32.33)^a^	140 (30.36)^b^	136 (29.69)^c^	0.023
Hypertension n (%)	161 (30.26)	132 (28.63)	135 (29.47)	0.351
Hyperlipidemia n (%)	167 (31.39)^a^	129(27.33)^b^	114(24.89)^c^	0.012
Smoking n (%)	128(24.06)	109(23.64)	107(23.36)	0.189
Atrial fibrillation n (%)	65 (12.21)	58 (12.58)	56 (12.22)	0.243
CAD n (%)	153 (28.95)^a^	132 (28.63)^b^	100 (21.83)^c^	**0.032**
PAD n (%)	35 (6.57)	28 (6.07)	26 (5.67)	0.089
MI rates n (%)	202 (37.9)^a^	157 (34.05)^b^	38 (8.29)^c^	**<0.001**
CAD-RADS	2.3±0.41^a^	2.2±0.39^a^	1.3±0.12^b^	**<0.001**
**Hematological variables**				
Fasting Glucose(mg/dl)	119.46±11.83	117.77±12.68	118.43±14.6	0.751
Creatinine (mg/dl)	0.98±0.45	0.99±0.25	1.08±0.41	0.127
Sodium (mmol/L)	139.56±2.86	137.62±3.8	138.18±4.58	0.891
Potassium (mmol/L)	4.51±0.34	4.50±0.44	4.43±0.52	0.493
ALT (U/L)	22.28±2.95	21.23±3.96	19.14±5.1	0.351
AST (U/L)	23.44±3.16	22.76±6.92	22.31±5.01	0.379
TSH(µIU/ml)	1.59±0.28	1.55±0.34	1.52±0.44	0.283
Hemoglobin (g/dl)	10.61±2.31	10.69±2.19	11.18±2.74	0.124
WBC count (x10^3^/µl)	9.81±5.00	9.88±5.47	9.68±4.32	0.551
Neutrophil count (×10^3^/μL)	7.04±2.45^a^	4.20±2.11^b^	4.13±2.14^b^	**<0.001**
Lymphocyte count (×10^3^/μL)	1.21±0.64^a^	1.39±0.94^b^	1.42±0.87^b^	**<0.001**
Monocyte (X10^3^/µl)	1.57±1.44	1.53±1.31	1.56±0.99	0.088
Platelet (x10^3^/µl)	339.27±81.13^a^	330.81±77.63^b^	329.64±81.9^b^	**<0.001**
LDL-cholesterol (mg/dl)	134.41 ± 37.8	123.6 ± 40.0	121.8±42.74	**<0.001**
HDL-cholesterol(mg/dl)	38.79±11.7	41.21±10.4	41.17±9.87	0.107
Triglyceride, (mg/dl)	221.8±31.31	187.5 ± 23.6	185.4 ± 22.4	**<0.001**
NT-proBNP (pg/ml)	47.45±11.23^a^	45.2±9.7^b^	43.8±9.3^c^	0.034
Troponin (ng/mL)	98.43±27.46^a^	56.24±24.78^b^	55.37±23.48^b^	**<0.001**
CRP (mg/L)	44.21±8.96^a^	22.41±7.41^b^	21.37±6.99^b^	**<0.001**
**Echocardiographic Findings**				
LVEF (%)	51.44±2.3	52.34±1.9	53.01±2.1	0.428
LVMI (g/m)	112.13±10.3	112.01±8.6	111.12±4.8	0.559
LA size (mm)	34.41±3.7	33.52±4.01	33.25±3.9	0.571
**Angiographic parameters**				
LAD lesion n(%)	220 (41.35)^a^	189 (40.99)^a^	110 (24.01)^b^	**<0.001**
CX lesion n(%)	200 (37.59)^a^	172(37.31)^a^	90 (19.65)^b^	**<0.001**
RCA lesion n(%)	155 (29.13)^a^	142 (30.80)^a^	52 (11.35)^b^	**<0.001**
Three vessel disease n(%)	30 (5.63)^a^	22 (4.77)^b^	6 (1.31)^c^	**<0.001**
**Medications**				
Acetylsalicylic acid	240 (45.11)^a^	205 (44.46)^a^	102 (22.27)^b^	**<0.001**
ACEI, ARB	162 (30.45)	142 (30.80)	140 (30.56)	0.153
Beta blocker	146 (27.44)	128 (27.76)	120 (26.20)	0.739
Statin	152 (28.57)^a^	123 (26.68)^a^	102 (22.27)^b^	**0.003**
Clopidogrel	33 (6.20)	27 (5.85)	25 (5.4)	0.224
Calcium channel blockers	61 (11.46)	52 (11.27)	50 (10.91)	0.786

^a,b,c^In Superscript explanations, the same letters show that there is no statistically significant difference between them, and different letters show that there is a statistically significant difference. Bold values indicate statistically significant p values (p<0.05). ACEI: angiotensin converting enzyme inhibitor; ALT: alanine Aminotransferase; ARB: angiotensin Receptor Blocker; AST: aspartate Aminotransferase; BMI: body mass index; CAD: coronary artery disease; CAD-RADS: coronary artery disease-reporting and data system; CRP: C-Reactive protein; CX: circumflex artery; HDL: high density lipoprotein; LA: left atrium; LAD: left anterior descending artery; LDL: low density lipoprotein; LVEF: left ventricular ejection fraction; LVMI: left ventricular mass index; MI: myocardial infarction; NT-proBNP: N-terminal pro-brain natriuretic peptide; PAD: peripheral artery disease; RCA: right coronary artery; TSH: thyroid stimulating hormone; WBC: white blood cell.

ROC analysis determined the optimal cut-off values for IBI and CAC scores in predicting MI ( Figure 1 ). The combination of both parameters demonstrated the highest levels of sensitivity and specificity in predicting MI in patients with elevated CAC and IBI scores ( Figure 2 ).

**Figure 1:** – ROC curves of IBI and CAC scores for predicting myocardial infarction in patients with coronary artery disease.

**Figure 2:** – ROC curve analysis of Coronary Artery Calcium (CAC) score and Inflammatory burden index (IBI) values, and the combination of CAC and IBI to predict myocardial infarction.

In multivariate logistic regression analysis, diabetes mellitus, hyperlipidemia, CAD, neutrophil count, LDL cholesterol, CRP, CAC score, and IBI were determined to be independent potential predictors of MI ( Table 3 ).

**Table 3:** – Univariate and multivariate regression analysis to identify independent predictors in myocardial infarction patients

Variable	Univariate Analysis		Multivariate Analysis	
	Odds Ratio (95% CI)	p-Value	Odds Ratio (95% CI)	p-Value
Hyperlipidemia	1.234 (0.998-1.379)	**0.017**	1.198 (0.745-1.543)	**0.047**
Diabetes mellitus	1.997 (0.982-2.134)	**0.003**	1.887 (0.996-2.245)	**0.032**
CAD	1.392 (1.048-1.592)	**0.040**	1.212 (0.896-1.531)	**0.046**
Statin use	1.028 (0.883-1.126)	**0.011**	0.952 (0.791-1.113)	0.143
Neutrophil count (x10^3^/µl)	1.124 (0.950-1.328)	**<0.001**	0.852 (0.657-1.145)	0.053
Lymp. Count (x10^3^/µl)	1.234 (0.997-1.438)	**<0.001**	0.763 (0.443-0.934)	0.159
Platelet (x1^03^/µl)	1.107 (0.761-1.278)	**<0.001**	0.972 (0.752-1.129)	0.093
LDL-cholesterol (mg/dl)	1.192 (0.837-1.349)	**<0.001**	1.292 (0.823-1.338)	**0.043**
HDL-cholesterol (mg/dl)	1.237 (0.973-1.425)	**0.048**	0.937 (0.738-1.173)	0.434
Triglyceride, (mg/dl)	1.058 (0.871-1.135)	**0.039**	0.932 (0.759-1.089)	0.537
Troponin (ng/mL)	1.473 (1.102-1.810)	**<0.001**	0.984 (0.749-1.227)	0.052
NT-proBNP (pg/ml)	1.342 (0.988-1.550)	**0.028**	0.892 (0.634-0.913)	0.376
CRP (mg/L)	1.627 (1.204-1.971)	**<0.001**	1.273 (1.104-1.426)	**0.027**
CAC score	2.014 (1.283-2.316)	**<0.001**	1.930 (1.254-2.364)	**0.018**
CAD-RADS	1.539 (1.011-1.679)	**<0.001**	1.430 (1.112-1.619)	**0.035**
IBI	2.234 (1.376-2.891)	**<0.001**	2.129 (1.637-2.461)	**<0.001**

Bold values indicate statistically significant p values (p<0.05). CAC: coronary artery calcium; CAD: coronary artery disease; CAD-RADS: coronary artery disease-reporting and data system; CRP: C-Reactive protein; HDL: high density lipoprotein; IBI: The inflammatory burden index; LDL: low density lipoprotein; Lymp: lymphocytes; NT-proBNP: N-terminal pro b-type natriuretic peptide.

Next, we assessed whether IBI and CAC scores were incrementally associated with the risk of MI in our long-term coronary artery disease follow-up cohort. Kaplan-Meier curves were used to analyze the differences between models with different IBI and CAC questions in predicting MACEs ( Figure 3 ). Patients with higher IBI and CAC scores were more likely to have MI than those with lower IBI and CAC scores (log-rank p < 0.001). According to our modeling system, a significantly higher MI rate was found in the Model 1 group than in the Model 2 group and in the Model 2 group than in the Model 3 group in this long-term follow-up cohort. After risk adjustment, 397 (32.14%) patients developed MI during follow-up ( Figure 3 ). Patients with high CAC (>102) and IBI (>128) were at higher risk of MI (p<0.001 for all). After risk adjustment, 202 patients (37.9%) experienced an MI within 3 years in the Model 1 patient group (odds ratio [OR] 1.484 [1.287-1.750]), 157 patients (34.05%) experienced an MI within 3 years in the Model 2 patient group ([OR] 1.184 [0.867-1.340]), and 38 patients (8.29%) experienced an MI within 3 years in the Model 3 patient group ([OR] 0.384 [0.237-0.431]).

**Figure 3:** – Myocardial infarction rate according to models.

## Discussion

In this retrospective study, we investigated the predictive value of IBI and CAC scores in identifying patients with a higher risk of MI (vulnerable plaque) in patients with plaque who underwent CT angiography due to chest pain. Our findings suggest that both IBI and CAC scores are significant predictors of MI for vulnerable plaque, with higher scores being associated with increased risk. These results underscore the potential utility of these indices in clinical practice for the early identification of high-risk patients and could aid in the refinement of therapeutic strategies.

The elevated levels of neutrophils, CRP, LDL cholesterol, triglycerides, NT-proBNP, and troponin observed in the MI group compared to the non-MI group are consistent with the well-established role of inflammation and lipid metabolism in the pathogenesis of atherosclerosis and subsequent plaque rupture.^13 , 14^The significantly higher IBI values in the MI group reinforce the hypothesis that systemic inflammation contributes to the development of acute coronary syndromes (ACS).^15^This finding aligns with previous research demonstrating that inflammatory biomarkers are powerful predictors of cardiovascular event.^16^

Similarly, the higher CAC scores in the MI group compared to the non-MI group confirm the role of coronary artery calcification as a marker of atherosclerotic plaque burden.^17^The association between higher CAC scores and an increased risk of MI has been extensively documented in the literature, where it is often used to stratify patients into different risk categories for future cardiovascular events.^18^The observation that patients in Model 1 (high IBI and CAC scores) had the highest rates of MI, followed by those in Model 2 and then Model 3, further supports the combined use of these indices for risk stratification.^19^

Coronary atherosclerosis manifests itself with the appearance of fatty streaks in the coronary arteries and other arterial beds in the very early stages of life. In fact, it is known today that atherosclerosis begins during fetal development, especially in fetuses of mothers with hypercholesterolemia.^20^In the development of atherosclerosis, first endothelial dysfunction, then leukocyte lipid and macrophage accumulation in the intima layer, then inflammation, and finally smooth muscle cell proliferation and migration from the medial layer, resulting in the formation of a fibrous cap on the fatty lesion. This lesion is now an irreversible complex lesion.^21^Since the publication of the original CAD-RADS classification, several prospective studies have provided evidence supporting the clinical utility of CCTA and the importance of CT findings in patients with suspected stable coronary artery disease. These include the PROMISE^22^and SCOT-HEART^23^studies, which demonstrated that CCTA is clinically useful as an alternative to functional testing or as an adjunct to standard of care. Based on these studies and multiple registries, the prognostic value of the CAD-RADS classification has been validated, with higher CAD-RADS scores being associated with increased risks of fatal and nonfatal MI.^9 , 24^Our study supports these studies and found that patients who underwent CAD-RADS had higher scores.

The ROC analysis provided additional evidence supporting the utility of IBI and CAC scores as predictors of MI. The cut-off values identified for IBI (128) and CAC (102) demonstrated good sensitivity and specificity, suggesting that these indices could serve as reliable markers for assessing the risk of MI in patients presenting with chest pain. Moreover, the multivariate logistic regression analysis identified diabetes, hyperlipidemia, coronary artery disease (CAD), neutrophil count, LDL cholesterol, CRP, CAC score, and IBI as independent predictors of MI, highlighting the multifactorial nature of this condition.^25^

One of the most significant findings of our study is the incremental value of combining IBI and CAC scores in predicting MACEs over the long term. Kaplan-Meier analysis showed a clear gradient in MI risk across the three models, with the highest risk in patients with both high IBI and CAC scores. This suggests that integrating inflammatory and calcification indices could provide a more comprehensive assessment of a patient’s cardiovascular risk, potentially guiding more personalized therapeutic interventions.^3 , 26^

The combination of systemic markers like CAC and IBI allows us to identify not just individual vulnerable plaques but ‘vulnerable patients’ who may be at higher risk for future cardiovascular events. The concept of the vulnerable patient represents a more holistic approach that encompasses systemic atherosclerosis burden, inflammatory status, and myocardial vulnerability, rather than focusing on a single plaque characteristic. In a patient with high CAC and IBI scores who has multiple non-obstructive plaques, the fact that one plaque leads to MI and another does not is likely the result of complex interactions between local factors (endothelial function, plaque morphology) and systemic factors (inflammation, coagulation tendency). Therefore, the use of CAC and IBI helps define the vulnerable patient rather than just specific vulnerable plaques.

Our study has several limitations that should be considered when interpreting the results. First, the retrospective design may introduce selection bias, and the exclusion of patients with incomplete data could have influenced our findings. Second, while our study population was relatively large, it was drawn from a single center, which may limit the generalizability of the results. Future studies should aim to validate these findings in larger, multi-center cohorts and explore the potential benefits of targeted therapies based on IBI and CAC scores.

Despite the limitations of our single-center retrospective design, these findings suggest that integrating inflammatory and calcification indices offers a more comprehensive assessment of cardiovascular risk. Further multi-center prospective research is needed to confirm these findings and to explore how these indices can be integrated into routine clinical practice to improve patient outcomes.

## Conclusion

In conclusion, our study supports the use of IBI and CAC scores as valuable tools for predicting MI in patients undergoing CT angiography. We identified optimal cut-off values of 128 for IBI and 102 for CAC score, which demonstrated good sensitivity and specificity in predicting future MI events. The combination of these indices appears to enhance risk stratification, with patients having both high IBI and high CAC scores showing significantly greater risk of MI during our 3-year follow-up period.

Our three-model system provides clinicians with a practical framework for identifying high-risk patients who may benefit from more intensive monitoring and preventive interventions. This approach enables us to identify not just vulnerable plaques but also ‘vulnerable patients’ who are more susceptible to cardiovascular events in the presence of systemic atherosclerosis and inflammation, thus providing a more holistic perspective in risk assessment. This approach could potentially allow for more tailored therapeutic strategies in high-risk patients, particularly those with evidence of both calcification and systemic inflammation.

### Highlights

1. The combination of IBI and CAC scores provides improved risk stratification for the prediction of future MI (vulnerable plaque) in patients with plaque detected by CT angiography.
2. Our study is the first to use the CAC score and IBI together.
3. The concept of the ‘vulnerable patient’, incorporating both CAC and IBI scores, provides a more comprehensive approach to cardiovascular risk assessment than focusing solely on individual plaque characteristics.
